# Clinical Implications of Cluster Analysis-Based Classification of Acute Decompensated Heart Failure and Correlation with Bedside Hemodynamic Profiles

**DOI:** 10.1371/journal.pone.0145881

**Published:** 2016-02-03

**Authors:** Tariq Ahmad, Nihar Desai, Francis Wilson, Phillip Schulte, Allison Dunning, Daniel Jacoby, Larry Allen, Mona Fiuzat, Joseph Rogers, G. Michael Felker, Christopher O’Connor, Chetan B. Patel

**Affiliations:** 1 Section of Cardiovascular Medicine, Department of Internal Medicine, Yale University School of Medicine, New Haven, Connecticut, United States of America; 2 Program for Translational Medicine, Yale University School of Medicine, New Haven, Connecticut, United States of America; 3 Division of Biomedical Statistics and Informatics, Mayo Clinic, Rochester, Minnesota, United States of America; 4 Duke Clinical Research Institute, Durham, North Carolina, United States of America; 5 Division of Cardiology, Department of Medicine, University of Colorado, Denver, Colorado, United States of America; 6 Division of Cardiology, Duke University Medical Center, Durham, North Carolina, United States of America; 7 Inova Heart and Vascular Institute, Falls Church, Virginia, United States of America; Scuola Superiore Sant'Anna, ITALY

## Abstract

**Background:**

Classification of acute decompensated heart failure (ADHF) is based on subjective criteria that crudely capture disease heterogeneity. Improved phenotyping of the syndrome may help improve therapeutic strategies.

**Objective:**

To derive cluster analysis-based groupings for patients hospitalized with ADHF, and compare their prognostic performance to hemodynamic classifications derived at the bedside.

**Methods:**

We performed a cluster analysis on baseline clinical variables and PAC measurements of 172 ADHF patients from the ESCAPE trial. Employing regression techniques, we examined associations between clusters and clinically determined hemodynamic profiles (warm/cold/wet/dry). We assessed association with clinical outcomes using Cox proportional hazards models. Likelihood ratio tests were used to compare the prognostic value of cluster data to that of hemodynamic data.

**Results:**

We identified four advanced HF clusters: 1) male Caucasians with ischemic cardiomyopathy, multiple comorbidities, lowest B-type natriuretic peptide (BNP) levels; 2) females with non-ischemic cardiomyopathy, few comorbidities, most favorable hemodynamics; 3) young African American males with non-ischemic cardiomyopathy, most adverse hemodynamics, advanced disease; and 4) older Caucasians with ischemic cardiomyopathy, concomitant renal insufficiency, highest BNP levels. There was no association between clusters and bedside-derived hemodynamic profiles (p = 0.70). For all adverse clinical outcomes, Cluster 4 had the highest risk, and Cluster 2, the lowest. Compared to Cluster 4, Clusters 1–3 had 45–70% lower risk of all-cause mortality. Clusters were significantly associated with clinical outcomes, whereas hemodynamic profiles were not.

**Conclusions:**

By clustering patients with similar objective variables, we identified four clinically relevant phenotypes of ADHF patients, with no discernable relationship to hemodynamic profiles, but distinct associations with adverse outcomes. Our analysis suggests that ADHF classification using simultaneous considerations of etiology, comorbid conditions, and biomarker levels, may be superior to bedside classifications.

## Introduction

Whereas acute decompensated heart failure (ADHF) has been treated by clinicians at least since the age of antiquity, descriptions of the condition have undergone several paradigm shifts as understanding of disease pathophysiology evolved [1]. Today, ADHF is viewed as a complex heterogeneous clinical syndrome, with classifications that rely heavily on non-specific descriptors such as left ventricular ejection fraction cut-points (HF with preserved vs. reduced ejection fraction) and hemodynamic profiles that are based on bedside assessments of cardiac output (“cold” vs. “warm”) and filling pressures (“wet” vs. “dry”)[2]. This construct theoretically allows for treatment decisions to be linked to patient categorization; nevertheless, there is increasing recognition that such subjective classifications are discordant with our current understanding of HF and fail to provide adequate phenotyping of this complex syndrome [3, 4]. Inadequate phenotyping of disease is also suggested as a major reason for a dismal record of drug development for ADHF [5].

As a result of these realizations, both European and North American Guidelines have expressed the need for a new taxonomy of disease on the basis of both clinical and molecular measures that may provide a more accurate HF disease classification, with the ultimate goal of enhancing diagnosis and treatment [2, 6]. Novel analytics like cluster analysis harness increased computing power, permitting us to use data-driven approaches to re-examine the phenotyping of complex diseases like ADHF [7]. Shah et al. recently used such an approach to describe three distinct subtypes of patients with stable HF with preserved ejection fraction [3]. Our group previously identified four distinct phenotypes of chronic systolic HF by applying cluster analysis to patients enrolled in the Heart Failure: A Controlled Trial Investigating Outcomes of Exercise Training (HF-ACTION) clinical trial [4]. However, prior examinations of HF phenotypes have excluded patients with ADHF and lacked information on invasive hemodynamics, limiting their ability to understand whether cluster analysis of objective clinical variables and directly measured hemodynamics result in clinically meaningful findings.

In order to explore this knowledge gap, in our current study, we applied cluster analysis to the pulmonary artery catheter (PAC) arm of the Evaluation Study of Congestive Heart Failure and Pulmonary Artery Catheterization Effectiveness (ESCAPE) trial of ADHF to describe patient characteristics and patterns of adverse clinical outcomes among the clusters. Furthermore, we examined the association of the clusters with clinically derived hemodynamic profiles. Our hypothesis was that applying advanced analytics to objective patient variables would yield patient groups that are superior to classifications derived at the bedside.

## Methods

### Study Population

Details outlining the design, rationale, and primary results of the ESCAPE trial have been previously published [8, 9]. Briefly, ESCAPE was a National Heart, Lung, and Blood Institute-sponsored multicenter trial designed to examine whether therapy guided by PAC invasive hemodynamic monitoring and clinical assessment improves patient outcomes more than therapy guided by expert clinical assessment alone in hospitalized HF patients. The ESCAPE trial was conducted in the United States and Canada at 26 sites between 2000 and 2003. Randomization required at least three months of symptoms, despite angiotensin-converting enzyme inhibitors and diuretics, left ventricular ejection fraction ≤30%, systolic blood pressure ≤125 mmHg, and at least one sign and one symptom of congestion. Exclusion criteria to minimize confounding comorbidities or urgent crossover included creatinine level >3.5 mg/dL, prior use of dobutamine or dopamine >3 μg/kg/min, and any prior use of milrinone during the index hospitalization. Of the 433 patients randomly assigned, 215 were assigned to the PAC arm. The primary results of the ESCAPE trial demonstrated that PAC use did not improve or worsen outcomes as assessed by the primary endpoint; these results have been published [9]. This study was approved by the Duke University School of Medicine Institutional Review Board, performed in accordance with the ethical guidelines of the Declaration of Helsinki, and all patients provided written informed consent.

### Assessment of Patient Variables

Data collected during patient assessments included patient demographics, admission signs and symptoms, physical examination findings, laboratory values, hemodynamics (in the PAC arm), complications, length of stay, and outcomes up to 180 days after discharge. We assessed physiologic parameters, natriuretic peptides, peak oxygen consumption, 6-minute walk distance, and the Minnesota Living with Heart Failure Questionnaire, without knowledge of group assignment. We acquired PAC hemodynamic data using standard methods, and only chose centers with expertise in invasive monitoring and clinical management of patients with HF; further training was provided to investigators. Cardiac output was measured by thermodilution in triplicate.

### Assessment of Hemodynamic Profiles

Blinded to randomization, investigators assessed the adequacy of peripheral perfusion with emphasis on warmth of extremities and proportional pulse pressure ≤25. Based on this evaluation, they classified subjects into one of four hemodynamic profiles according to adequacy of cardiac output (“warm” or “cold”) and increase in left-sided filling pressures (“wet” or “dry”). Specific criteria for when to classify the patient as “wet” or “cold” (i.e., at what estimated pulmonary capillary wedge pressure [PCWP] or cardiac output) was left to the investigator’s discretion.

### Goals of Treatment and Clinical Endpoints

The treatment goal in the clinical assessment group was resolution, measured as changes from baseline, of clinical signs and symptoms of congestion, particularly jugular venous pressure elevation, edema, and orthopnea. The treatment goal in the PAC group was the same, with the addition of a PCWP of 15 mmHg and a right atrial pressure of 8 mmHg. Therapy was adjusted in both groups to avoid progressive renal dysfunction or symptomatic systemic hypotension. Clinical endpoints included all-cause mortality; cardiovascular death; rehospitalization; HF rehospitalization; and a composite endpoint of all-cause mortality, cardiac rehospitalization, and cardiac transplant.

### Statistical Analysis

Hierarchical cluster analysis is a process used to determine similar patients groups based on the combined values of their measured characteristics, without knowledge of outcomes. We used Ward’s Minimum-Variance Method to cluster patients together based on 14 baseline candidate variables. This method calculates the distance between any two clusters, where a cluster could be an individual patient or group of patients already clustered together and where distance is defined as a function of squared Euclidean distance. If that distance is sufficiently small than those clusters are grouped together; when the distance between clusters gets too large a new grouping is started and the process continues… We selected the following 14 candidate variables measured at baseline that represented key objective characteristics of 162 patients with HF: age, sex, race, systolic blood pressure, left ventricular ejection fraction, heart rate, ischemic etiology of HF, blood urea nitrogen, creatinine, B-type natriuretic peptide (BNP), right atrial pressure, PCWP, and cardiac index. These variables were included in the cluster analysis, and four clusters were pre-specified, since this matches the number of HF hemodynamic profiles.

The four patient clusters were compared for association with the four clinically determined hemodynamic profiles of “warm/wet,” “cold/wet,” “cold/dry,” or “warm/dry” using χ^2^ analysis. Comparison of treatment goal resolution via changes from baseline to discharge of the variables listed above in the PAC group were conducted using Wilcoxon Signed Rank test within each of the four patient clusters. Kaplan-Meier plots describe survival of clinical outcomes by cluster membership and the association between cluster membership and clinical outcomes was assessed using Cox proportional hazards regression. We used likelihood ratio tests for nested survival models to determine if the addition of cluster membership to a Cox regression model already containing hemodynamic profiles would provide better risk predictability than the Cox regression model containing hemodynamic profiles alone. All analyses were performed using SAS version 9.4 (SAS Institute, Inc. Cary, NC, USA) and R 2.15.3 (R Development Core Team, Vienna, Austria). A p-value ≤0.05 was considered statistically significant for all analyses.

## Results

Baseline characteristics of the patients in the four clusters are described below, and are listed in Table 1. Comparisons across clusters was conducted using the non-parametric Kruskal-Wallis test for continuous variables and Chi-square tests for categorical variables.

**Table 1:** Baseline Characteristics According to Patient Cluster*.

Characteristic	Cluster 1 (n = 75)	Cluster 2 (n = 33)	Cluster 3 (n = 29)	Cluster 4 (n = 25)	p-value^†^
Age, years	58 (46–67)	52 (44–59)	51 (42–57)	69 (59–79)	<0.001
Female, %	5	100	3	24	<0.001
Race					<0.001
White, %	87	52	0	76	
Minority, %	12	45	100	24	
Ischemic etiology, %	65	30	10	84	<0.001
LVEF, %	20 (15–23)	20 (15–25)	15 (13–18)	20 (19–25)	0.001
BMI, kg/m^2^	29 (25–34)	26 (23–36)	28 (25–30)	24 (22–26)	0.013
Edema, %	72	56	79	60	0.145
Symptom score	40 (30–60)	44 (30–60)	35 (20–50)	50 (34–60)	0.295
MLHF score	78 (68–87)	76 (63–95)	83 (72–89)	74 (64–78)	0.212
Orthopnea, %	88	85	86	76	0.529
SBP, mmHg	100 (90–111)	109 (97–120)	110 (103–124)	100 (90–114)	0.005
DBP, mean	65 (60–70)	66 (56–70)	76 (68–85)	59 (55–70)	<0.001
Atrial fibrillation, %	44	15	7	24	<0.001
Angina pectoris, %	36	21	21	44	0.127
Prior CABG, %	32	15	7	64	<0.001
COPD, %	13	9	24	24	0.235
Depression, %	21	27	14	20	0.634
Diabetes, %	39	30	25	40	0.493
Hypertension, %	43	49	62	28	0.084
ICD, %	33	12	28	28	0.156
CVA, %	12	6	3.4	8	0.601
Peak VO_2_, mL/kg/min	10.4 (8.0–11.9)	9.1 (7.3–10.6)	8.7 (7.6–9.3)	9.0 (7.6–10.4)	0.517
RAP, mmHg	13 (8–18)	11 (6–14)	17 (13–22)	14 (9–20)	0.005
PCWP, mmHg	27 (19–34)	22 (15–28)	32 (28–38)	23 (20–27)	<0.001
Cardiac index, L/min/m^2^	1.9 (1.6–2.3)	2.0 (1.5–2.2)	1.6 (1.2–2.2)	1.8 (1.6–2.5)	0.120
Sodium, mEq/L	137 (134–139)	138 (136–139)	137 (136–139)	136 (134–138)	0.403
BUN, mg/dL	29 (20–41)	20 (12–26)	29 (23–41)	80 (47–98)	<0.001
Creatinine, mg/dL	1.4 (1.2–1.6)	0.9 (0.9–1.2)	1.4 (1.3–1.8)	2.5 (2.1–3.1)	<0.001
BNP, pg/mol	469 (174–963)	489 (183–860)	877 (89–1391)	1398 (518–4513)	0.001

BMI indicates body mass index;

BNP, B-type natriuretic peptide;

BUN, blood urea nitrogen;

CABG, coronary artery bypass grafting;

COPD, chronic obstructive pulmonary disease;

CVA, cerebrovascular accident;

DSP, diastolic blood pressure;

ICD, implantable cardioverter defibrillator;

LVEF, left ventricular ejection fraction;

MLHF, Minnesota Living With Heart Failure Questionnaire;

PCWP, pulmonary capillary wedge pressure;

RAP, right atrial pressure;

SBP, systolic blood pressure;

VO_2_, oxygen consumption

*Values are median (interquartile range), or %.

^†^p-values for the comparisons of variables across clusters.

### Cluster 1 (n = 75)

Cluster 1 was the largest cluster, with more than twice the number of patients than any other cluster. Patients were primarily Caucasian males (87%) with ischemic cardiomyopathy (65%). They tended to have multiple comorbid conditions. Specifically, they had the highest median body mass index, prevalence of atrial fibrillation, implantable cardioverter defibrillator use, and prior cerebrovascular accident. They had the second highest prevalence of angina, diabetes, depression, and prior coronary artery bypass grafting. This cluster had the second lowest symptom and Minnesota Living With Heart Failure Questionnaire scores, signifying poor quality of life. Importantly, there was evidence of renal dysfunction, with median creatinine levels of 1.4 mg/dL. These patients had abnormal hemodynamics at baseline, with only Cluster 3 having more adverse values: median PCWP was 27 mmHg and cardiac index was 1.9 L/min/m^2^. Interestingly, Cluster 1 patients had the lowest median BNP levels (469 pg/mol) and the highest peak oxygen consumption (10.4 mL/kg/min).

### Cluster 2 (n = 33)

Cluster 2 patients were all females (100%) who tended to have nonischemic cardiomyopathy (70%); there was an equal mix of Caucasians and minorities. This cluster had the second highest symptom and Minnesota Living With Heart Failure Questionnaire scores, signifying better quality of life. This group was the least likely to have peripheral edema on admission and had the lowest rates of implantable cardioverter defibrillator implantation. Cluster 2 was the only cluster with normal median renal function, as gauged by serum creatinine at baseline, and there was a low prevalence of comorbid conditions, with only Cluster 3 having lower rates. These patients had the most favorable hemodynamics on admission, with a median PCWP of 22 mmHg and a cardiac index of 2.0 L/min/m^2^. They had the second lowest median BNP levels (489 pg/mol) and second highest peak oxygen consumption (9.1 mL/kg/min).

### Cluster 3 (n = 29)

Cluster 3 patients were primarily African American males. These patients were the youngest group (median age = 51), and had nonischemic cardiomyopathy (90%) with the lowest median left ventricular ejection fraction (15%). They had the most adverse symptoms and poorest quality of life scores. They had the lowest rates of most comorbid conditions, except for hypertension, for which they had the highest prevalence. Subjects in this cluster had the most adverse hemodynamics on admission, with a median PCWP of 33 mmHg and a cardiac index of 1.6 L/min/m^2^. They had the second highest median BNP levels (877 pg/mol) and the lowest peak oxygen consumption (8.7 mL/kg/min).

### Cluster 4 (n = 25)

Cluster 4 was comprised of the oldest patients, who were primarily Caucasian (76%) males (76%) with ischemic cardiomyopathy (84%). They tended to have the least adverse symptoms and best quality of life scores. They had a heavy burden of comorbid illnesses, with the highest prevalence of angina, prior coronary artery bypass grafting, chronic obstructive pulmonary disease, and diabetes. This group’s defining feature appeared to be concomitant renal dysfunction, with a median blood urea nitrogen of 80 mg/dL and creatinine of 2.5 mg/dL. Cluster 4 patients had the second lowest median PCWP (23 mmHg) and cardiac index (1.8 L/min/m^2^), and the highest median BNP levels (1398 pg/mol).

### Changes with Therapy According to Cluster

Table 2 demonstrates therapies in key disease variables according to cluster. Comparison of change from baseline to follow-up in these HF parameters was done using the non-parametric Wilcoxon Signed Rank test for paired data. There were statistically significant differences in almost all parameters shown in each cluster. In Clusters 1 and 3, there were no significant changes in median serum creatinine (p = 0.365 and p = 0.66, respectively). Increase in median cardiac index was borderline significant in Cluster 4 (p = 0.054). Follow-up hemodynamics (i.e., right atrial pressure, PCWP, cardiac index) were not significantly different across clusters, but the greatest improvements were noted in Cluster 3 patients, who had a median right atrial pressure decrease of 10 mmHg, a PCWP decrease of 16 mmHg, and a cardiac index increase of 0.6 L/min/m^2^. The lowest degree of improvement was seen in Cluster 4 patients who had a median right atrial pressure decrease of 4 mmHg, a PCWP decrease of 5 mmHg, and a cardiac index increase of 0.3 L/min/m^2^. Despite equalization in hemodynamics, there were significant differences in prognostic biomarker levels. Specifically, median discharge BNP levels ranged from 232–632 pg/mL and creatinine levels from 1.1–2.0 mg/dL. The highest discharge BNP and creatinine levels were in Cluster 4 (632 pg/mL and 2.0 mg/dL, respectively), whereas the lowest were in Cluster 2 (232 pg/mL and 1.1 mg/dL, respectively), reflecting the risk of adverse clinical outcomes.

**Table 2:** Changes in HF Parameters from Admission to Discharge According to Patient Cluster*.

	Cluster 1				Cluster 2				Cluster 3				Cluster 4			
Variable	Baseline	Follow-up	Change	p-value	Baseline	Follow-up	Change	p-value	Baseline	Follow-up	Changes	p-value	Baseline	Follow-up	Change	p-value
RAP^†^ mmHg	13 (8, 18)	8 (5, 12)	-5 (-9, 0)	<0.001	11 (6, 14)	7 (3,11)	-3 (-7,-1)	<0.001	17 (13,22)	8 (5,10)	-10 (-14,-5)	<0.001	14 (9,20)	8 (5,11)	-4 (-7,0)	0.007
PCWP^‡^ mmHg	27 (19, 34)	17 (12, 21)	-11 (-18, -3)	<0.001	22 (15,28)	15 (10,18)	-8 (-14,2)	<0.001	32 (28,38)	15 (12,19)	-16 (-23,-9)	<0.001	23 (20–27)	17 (16,20)	-5 (-10,0)	0.001
CI, L/min/m^2^	1.9 (1.6, 2.3)	2.2 (1.9, 2.5)	0.4 (-0.1, 0.8)	<0.001	2 (1.5,2.2)	2.4 (1.9,2.7)	0.4 (0.1,0.7)	0.005	1.6 (1.2,2.2)	2.2 (1.9,2.4)	0.6 (0.3,1.0)	<0.001	1.8 (1.6–2.5)	2.4 (1.9,2.8)	0.3 (0,0.9)	0.054
BNP, pg/mol	469 (174, 963)	344 (138, 681)	-79 (-362,2)	0.095	489 (183, 860)	232 (135, 610)	-71 (-269,15)	0.04	877 (89, 1391)	319 (147, 662)	-335 (-741,30)	0.004	1398 (518, 4513)	632 (308,1727)	-365 (-1373,-9)	0.003
Cr, mg/dL	1.4 (1.2, 1.6)	1.4 (1.2–1.7)	0 (-0.2,0.2)	0.365	0.9 (0.9,1.2)	1.1 (0.8,1.5)	0 (-0.1,0.2)	0.045	1.4 (1.3,1.8)	1.3 (1.1,1.7)	-0.1 (-0.3,0.3)	0.66	2.5 (2.1–3.1)	2 (1.8,2.5)	-0.4 (-0.8,0.2)	0.023

Cr indicates creatinine; All other abbreviations can be found in Table 1.

*Values are median interquartile range.

^†^Change between baseline and optimal measurement just prior to PAC removal.

^‡^Change between baseline and 1 month.

### Association with Hemodynamic Profiles

**Fig 1:**
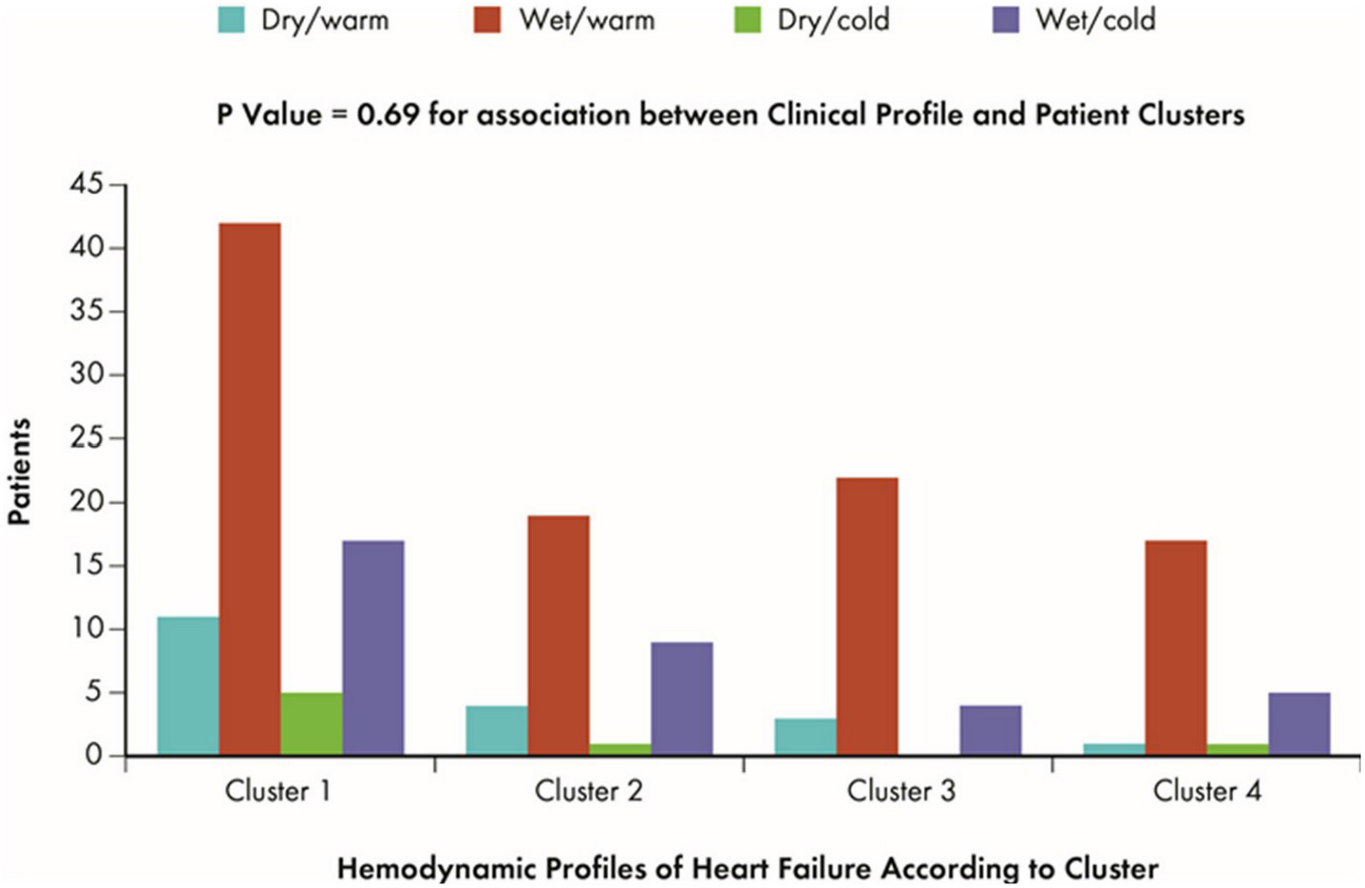
Patient Clusters and Hemodynamic Profiles. Fig shows frequency of patients for each hemodynamic profile of HF according to the four clusters identified in ESCAPE study; p-value demonstrates the association between clusters and hemodynamic profiles.

We examined relationships between the four identified clusters and bedside investigator-determined hemodynamic profiles of HF (Fig 1). The “wet/warm” profile of HF was most common in all clusters, ranging from 56% of Cluster 1 patients to 76% of Cluster 3 patients. The “wet/cold” profile was second most common, ranging from 14% of Cluster 3 to 27% of Cluster 2. The “dry/warm” profile was present in 4.2% of Cluster 4 to 15% of Cluster 1. The “dry/cold” profile was least common, with only seven patients fitting this profile. We found no statistical evidence of an association between our four clusters and the four hemodynamic profiles of HF (p = 0.70).

### Association with Clinical Outcomes

**Fig 2:**
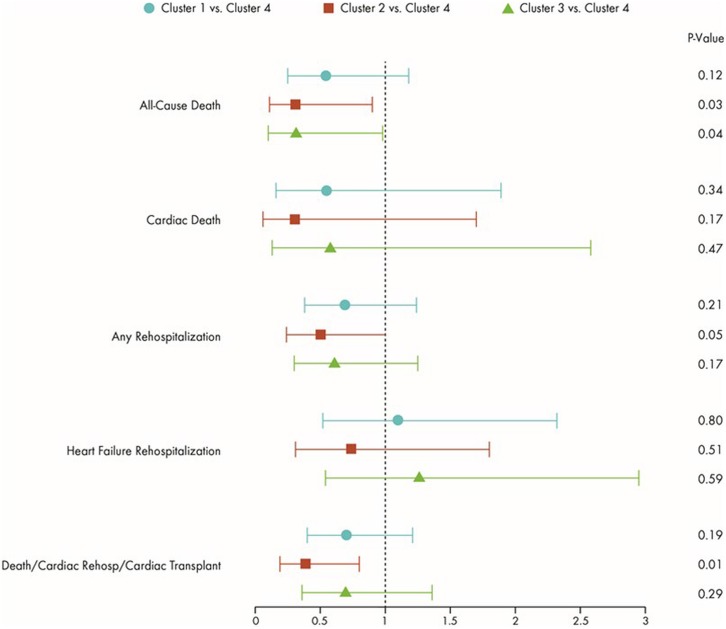
Risk of Clinical Events According to Cluster (Compared with Cluster 4). Symbols represent Hazard Ratios and 95% Confidence Intervals.

**Fig 3:**
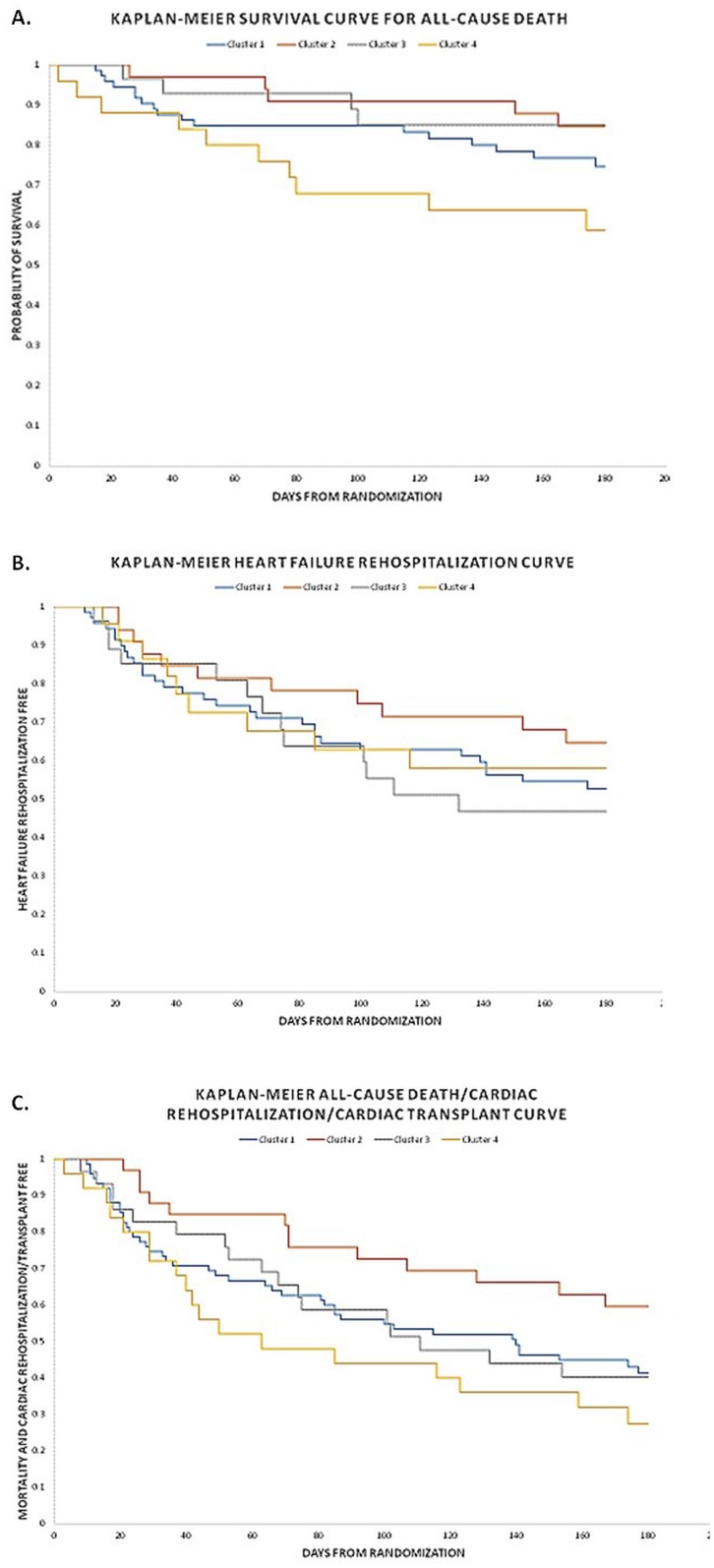
Kaplan Meir Curves According to Patient Cluster. This Fig shows time-to-outcome according to patient clusters for: (a) all-cause mortality; (b) Heart Failure hospitalization; and (c) composite endpoint of all-cause mortality, cardiac hospitalization, and cardiac transplant.

There was no evidence of an association between hemodynamic profiles and any clinical outcome (p>0.5, all). Fig 2 shows differences in clinical outcomes risk according to patient clusters from the ESCAPE trial, with Cluster 4 (highest risk) as the comparator group. There was a lower risk of all-cause death in Clusters 1–3, but all-cause death reached statistical significance in the case of Cluster 2 (p = 0.03) and Cluster 3 (p = 0.04). Notably, despite having the most adverse hemodynamics, Cluster 3 had similar risk of all-cause death as Cluster 2 (which had the most favorable hemodynamics). Cluster 2 had a 60% less chance of death/cardiac rehospitalization/transplant when compared with Cluster 4 (p = 0.01). Also, risk of any hospitalization tended to be statistically lower for patients in Cluster 2 when compared with Cluster 4 (p = 0.05). Interestingly, despite far lower rates of mortality, hazard ratios for risk of HF rehospitalization was similar for Cluster 3 when compared with Cluster 4, (hazard ratio [HR] = 1.26; 95% confidence interval [CI] 0.54–2.95; p = 0.60). Fig 3 demonstrates Kaplan-Meier survival curves for the endpoints of all-cause death, HF rehospitalization, and the composite endpoint of all-cause death, cardiac rehospitalization, and transplant. As displayed, patients in Cluster 2 had the lowest risk for all outcomes. Cluster 4 patients appeared to be at highest risk for all outcomes except for HF rehospitalization, where it seemed that Cluster 3 might be at higher risk.

### Comparison of Predictive Value of Patient Clusters to Hemodynamic Profiles

The additional prognostic benefit of adding cluster information to prediction models containing hemodynamic profiles is shown in Table 3. As shown, cluster information emerged as an independent predictor of all-cause death above and beyond hemodynamic profiles (p = 0.03). We found a trend towards significant benefit of cluster information for prediction of all-cause rehospitalization (p = 0.08). Lastly, we showed that addition of cluster information to a model containing hemodynamic profiles significant improved model performance for prediction of all-cause death/cardiac rehospitalization/cardiac transplant (p = 0.05).

**Table 3:** Additional Predictive Benefit of Clusters to Models Containing Hemodynamic Profiles.

Clinical Outcome	Model	AIC	Likelihood Ratio	Diff LR	p-value
All-Cause Death	Hemodynamic Profile	355.17	2.93	7.35	0.03
	Hemodynamic Profile + Cluster	353.83	10.28		
Cardiovascular Death	Hemodynamic Profile	160.34	2.60	2.23	0.19
	Hemodynamic Profile + Cluster	164.10	4.83		
All-Cause Rehospitalization	Hemodynamic Profile	811.50	1.41	4.777	0.08
	Hemodynamic Profile + Cluster	812.72	6.18		
Heart Failure Rehospitalization	Hemodynamic Profile	594.37	1.70	2.029	0.21
	Hemodynamic Profile + Cluster	598.34	3.72		
Death/Cardiovascular Rehospitalization/Cardiac Transplant	Hemodynamic Profile	1013.63	0.05	6.141	0.05
	Hemodynamic Profile + Cluster	1013.49	6.19		

## Discussion

Using a non-hypothesis driven statistical methodology that clusters patients according to similar clinical variables and hemodynamic measures in the invasive arm of the ESCAPE trial, we identified four clinically relevant phenotypes of ADHF. Patients within each cluster have previously described disease characteristics and distinct clinical trajectories. We noted no discernable relationship with clinically used hemodynamic profiles of HF. Furthermore, clusters, but not hemodynamic profiles, showed independent prognostic value. These findings raise the possibility that a data-driven approach to classification of HF might capture disease heterogeneity more appropriately, allowing for more efficacious use and testing of therapeutic interventions.

Our study follows recent efforts that have used advanced analytics to improve the accuracy of HF classification. Our group previously applied cluster analysis to baseline clinical variables from 1619 participants enrolled in the HF-ACTION study of exercising training in ambulatory patients with systolic HF, yielding four distinct phenotypes [4]. Shah et al. applied unbiased hierarchical cluster analysis to phenotypic data from 397 patients with HF with preserved ejection fraction, identifying three distinct groups that differed markedly in clinical characteristics, cardiac structure/function, invasive hemodynamics, and outcomes.^7^ Cluster analysis has also been applied to similarly heterogeneous syndromes such as chronic obstructive pulmonary disease, Parkinson’s disease, and human encephalitis, leading to new insights about disease pathophysiology [10–12].

We believe our findings are important for a several reasons. Perhaps most significantly, we found that applying an agnostic data-driven approach to commonly measured variables in advanced HF patients can yield clinically recognizable and previously described phenotypes of the disease that have varied clinical courses. The clusters derived using these methods showed no relationship to universally used bedside methods hemodynamic profiles for prediction of adverse outcomes.

Cluster 1 was comprised of Caucasian males with ischemic cardiomyopathy and high rates of comorbid conditions; this group of patients has been previously described in registry data [13]. Given the degree of comorbidities seen in this patient population, reducing adverse outcomes in this group may require a multipronged therapeutic approach, rather than simply focusing on cardiac-related disease features [14, 15].

Cluster 2 was comprised largely of females with non-ischemic cardiomyopathy and relatively low risk of adverse events. Previous clinical trial and registry data have indicated that women with HF have better age-adjusted survival rates than men, which has been postulated to result from a variety of causes, ranging from differences in biology to variability in psychosocial risk [16]. In our study, we noted a higher proportion of non-ischemic cardiomyopathy among women, low rates of comorbid conditions, and a clear signal towards more favorable hemodynamics. Given the low percentage of women in clinical trials, the true efficacy of guideline-recommended therapies in this group remains unclear. We postulate that in the setting of isolated systolic HF without severe hemodynamic compromise or comorbidity burden, this cluster may be most appropriately targeted for reverse remodeling using treatments like intensive neurohormonal antagonism and cardiac resynchronization therapy [17].

Cluster 3 was comprised almost entirely of African American males with non-ischemic cardiomyopathy; these patients were the youngest, but had the most adverse hemodynamics. Several prior studies have shown that African American patients with advanced HF have a different clinical profile than Caucasian patients: they are more likely to be younger, have nonischemic cardiomyopathy, and higher rates of rehospitalization [18]. These similarities suggest that the noted differences in disease characteristics and outcomes for this cluster might represent a unique HF phenotype, presenting us with an opportunity to target both underlying biology and environmental factors [19]. The dissociation between increased risk of HF hospitalization in the setting of relatively lower risk of death, might signal that this patient population could also benefit from disease management strategies aimed at closely monitoring for signs of volume overload [20]. Furthermore, our finding that Cluster 3 had the most deranged hemodynamics without hypotension, and had a high incidence of underlying hypertension, may represent an important treatment target. Vasodilator therapy has long been utilized to treat HF, but it remains unclear what population might benefit most from this therapy [21]. The African-American Heart Failure Trial (A-HeFT) demonstrated improved outcomes in African American patients with the use of hydralazine and nitrates; it would be intriguing to explore whether this was only beneficial to patients with Cluster 3 characteristics. There is potential to test several other novel vasodilators in this patient population such as Seralaxin, synthetic natriuretic peptides, and Clevidipine.

Cluster 4 was comprised of older patients with ischemic cardiomyopathy and concomitant renal failure. These patients had the most adverse outcomes, since they had higher risk characteristics, as described previously in the ESCAPE risk model and discharge score (i.e., increased age, renal failure, and high BNP levels at discharge)[22]. Since clinical trials in HF generally exclude this high-risk patient profile, the balance between therapeutic benefit and harm remains unclear, since it is possible these patients may have disease that is advanced beyond the benefit of our current strategies. Further studies are required to determine if risk-based resource allocation of advanced therapies such as inotropes, dialysis, and mechanical circulatory support lead to improved outcomes when compared with a strategy focused on physical and psychosocial symptom relief, attention to spiritual concerns, and advanced care planning [23].

In order to classify and treat ADHF, current guidelines recommend the use of hemodynamic profiles based on bedside assessments of congestion and perfusion [2]. The four recommended profiles—wet/cold, wet/warm, dry/cold, and dry/warm—were adapted to advanced HF from Forrester-Diamond classifications of congestion and perfusion estimates in acute myocardial infarction [24]. Furthermore, treatment options are recommended based on each profile without trial-based evidence in support of such an approach [8, 25]. Our results add to the evidence to the contrary, especially as we found no differences in invasive hemodynamics between clusters at discharge, but varied risks of adverse clinical outcomes. The findings presented here suggest that a data-driven approach—one that is increasingly possible with ubiquitous use of electronic medical records and improved computing—can classify patients more appropriately than bedside classifications.

Our findings also highlight the extreme heterogeneity that exists within advanced systolic HF, raising the question as to whether the efficacy of a single therapeutic approach can be tested appropriately in a population with such disparate characteristics and outcomes. For example, based on the results of the ESCAPE trial, the authors surmised that “*addition of the PAC to careful clinical assessment increased anticipated adverse events*, *but did not affect overall mortality and hospitalization”*[9]. We would argue that a more homogenous population of patients would be required to definitively test this hypothesis; for example, the competing risks of concomitant renal failure and comorbid conditions in Cluster 4 might make the achievement of optimal hemodynamics less important than in Cluster 3, where adverse hemodynamics and hypertension appeared to play a more central role in the clinical picture. Physicians involved in this study appeared to have acted along this preconceived notion by not enrolling patients with more advanced disease and concomitant renal failure into the trial [26]. As a result, it is possible that the optimal population, in whom PAC-guided therapy might be efficacious, still remains unknown.

Our study had several limitations. First, we are not attempting to propose a new classification of ADHF; rather, our motivation for this analysis is to demonstrate that the application of advanced analytic methods to multidimensional patient data can yield clinically relevant patient groupings that may lead to improvements HF classification. We are hopeful that these efforts will motivate more comprehensive endeavors to phenotype HF integratively through objective genetic, genomic, biochemical, cellular, physiological, and clinical measures of disease. Second, our clustering algorithm yielded results based on patients with complete data from the invasive arm of the ESCAPE trial. Use of more comprehensive data, as well as a greater number of objective patient variables, might have yielded different results. As a result of this unique dataset (with invasive hemodynamics and concomitant BNP data), we were unable to provide a validation analysis. Finally, our population was comprised of patients with advanced systolic HF who were enrolled in the ESCAPE trial, a trial that was completed a decade back; our patients and our findings is unlikely to generalizable to current patients, especially given the dramatically reduced rates in use of PACs.

## Conclusion

We used a non-hypothesis driven statistical approach that clustered patients according to similar clinical variables, BNP levels, and PAC measures to identify four clinically relevant phenotypes of ADHF. We found that patients within each cluster fit characteristics of previously described subgroups of HF, but demonstrated substantial differences in key disease characteristics and clinical outcomes. There were no associations between clusters and clinically determined hemodynamic profiles of ADHF. Cluster information, but not hemodynamic profile information, had independent prognostic value. Our results highlight the high degree of heterogeneity within the syndrome of ADHF, and the possibility that classification can be enhanced by novel multidimensional categorization approaches.
